# Determining Host Metabolic Limitations on Viral Replication via Integrated Modeling and Experimental Perturbation

**DOI:** 10.1371/journal.pcbi.1002746

**Published:** 2012-10-18

**Authors:** Elsa W. Birch, Nicholas A. Ruggero, Markus W. Covert

**Affiliations:** 1 Chemical Engineering, Stanford University, Stanford, California, United States of America; 2 Bioengineering, Stanford University, Stanford, California, United States of America; The Pennsylvania State University, United States of America

## Abstract

Viral replication relies on host metabolic machinery and precursors to produce large numbers of progeny - often very rapidly. A fundamental example is the infection of *Escherichia coli* by bacteriophage T7. The resource draw imposed by viral replication represents a significant and complex perturbation to the extensive and interconnected network of host metabolic pathways. To better understand this system, we have integrated a set of structured ordinary differential equations quantifying T7 replication and an *E. coli* flux balance analysis metabolic model. Further, we present here an integrated simulation algorithm enforcing mutual constraint by the models across the entire duration of phage replication. This method enables quantitative dynamic prediction of virion production given only specification of host nutritional environment, and predictions compare favorably to experimental measurements of phage replication in multiple environments. The level of detail of our computational predictions facilitates exploration of the dynamic changes in host metabolic fluxes that result from viral resource consumption, as well as analysis of the limiting processes dictating maximum viral progeny production. For example, although it is commonly assumed that viral infection dynamics are predominantly limited by the amount of protein synthesis machinery in the host, our results suggest that in many cases metabolic limitation is at least as strict. Taken together, these results emphasize the importance of considering viral infections in the context of host metabolism.

## Author Summary

Viral infection is a serious problem with relatively few known solutions. Much of the complexity of viral infection is contributed by the host's own resources that the virus commandeers. Viruses lack the machinery and precursors required to replicate, and thus may be considered metabolic products of their host. Our goal is a systems-level understanding of host-viral metabolic interaction via computational tools and quantitative dynamic measurements. Here we present an integrated model of T7 phage viral replication and host *E. coli* metabolism that predicts phage production changes across media conditions and provides insight into the underlying limiting factors in T7 replication. The model simulations, supported by our experimental measurements, highlight the role of host metabolism in determining the dynamics of viral infection.

## Introduction

Any virus is necessarily a metabolic product of its host, since viruses lack the macromolecule machinery and small molecule precursors required to replicate. This dependence has been underscored by recent screens to determine the host genes required for viral infection in a variety of species. The published sets of host-gene viral dependencies have consistently included metabolic genes - both enzymes and regulators - in systems ranging from phages T7 and lambda, to the human viruses HIV and influenza [1]–[8]. In complementary findings, some bacterial viruses have recently been shown to encode components as well as direct modifiers of host metabolic machinery [9], [10]. Taken together, these studies emphasize the need to understand viral infection in the context of host metabolism [11].

Viral host dependency screens are useful for identifying individual host genes involved in the metabolic interplay of viral infection; however, studying any of these single points of connection is likely to reveal a complex network of host-viral interactions [12]. Understanding infection as a highly integrated system is therefore necessary to predict the outcome of viral infection following perturbations, such as changes to the host nutritional environment. Similarly, metabolism is a deeply interconnected network, and viral infection represents a dynamic perturbation of it. Achieving a systems-level understanding of host-viral metabolic interaction therefore requires, a strong set of computational tools coupled with quantitative dynamic measurements.

Given the challenge presented by developing such modeling tools and making the needed measurements, bacteria and their viruses, particularly *E. coli* and certain of its bacteriophages, are favorable candidate model systems for building a systems-level understanding of infection. These systems have a long history of study, individually and together, and as a result are associated with a wealth of well-established observations and experimental protocols. Additionally, the host-viral dependency screens involving *E. coli* identified sets of genes whose products were far better characterized and annotated than in any other screen [1], [2]. These systems also have industrial relevance: threatening large-scale cultures [13], and alternately providing highly specific disinfection tools [14].

**Figure 1:**
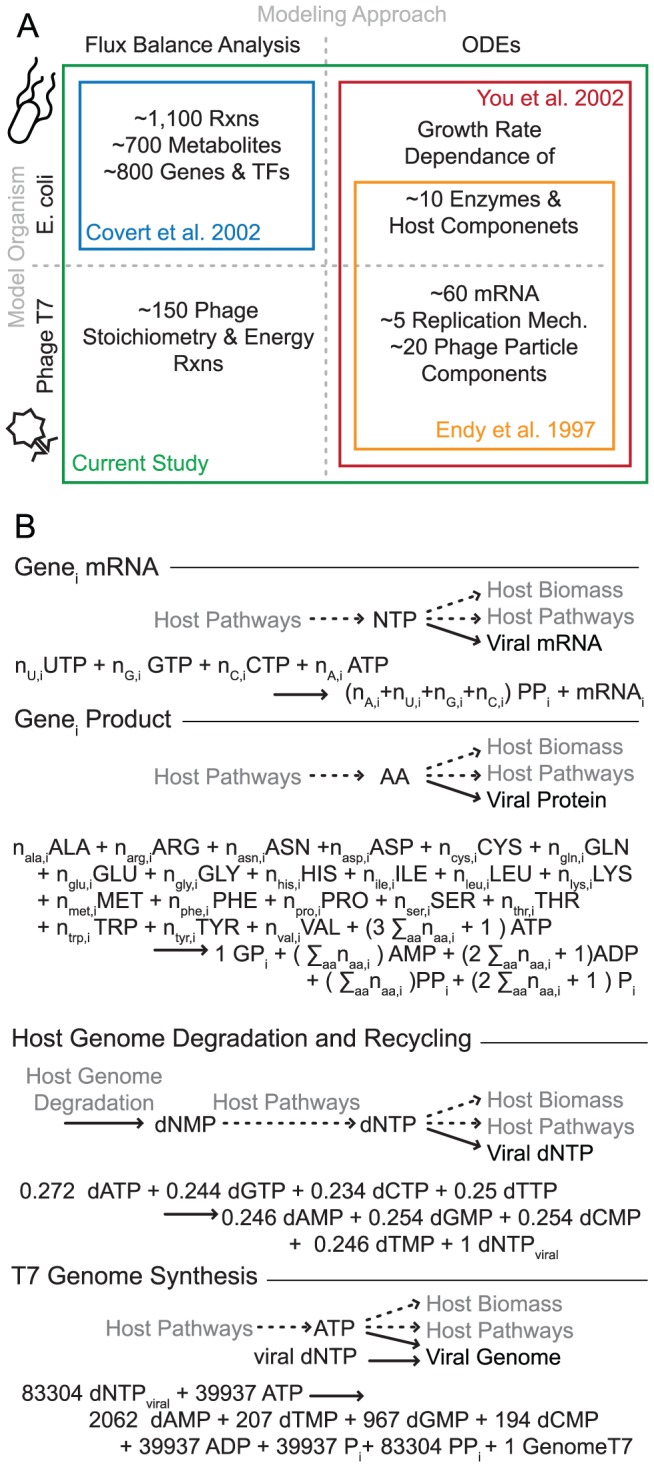
Model approaches, scopes, and additions used in the current integration. (A) The computational methods and the organisms represented by previous modeling efforts that are combined in this study. (B) The additional reactions constructed in this study for the purpose of translating T7 ODE reaction rates into host metabolite use. Shown at the top for each category is a schematic of metabolite connections to host metabolism, and under it the full stoichiometric reaction, which may be a formula based on nucleotide or amino acid sequence (the gene designations 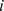 taking both decimal and integer values in correspondence with the naming of T7 genes [36], a total of n = 59 included). Assumptions made in formulating the reactions are expanded in Methods and SI, and the metabolite abbreviations used are consistent with the FBA model definition.

Critically, *E. coli* and its phage are sufficiently understood to enable the construction of predictive computational models. Phage T7 replication has been described with structured ordinary differential equations (ODEs), that account for the dynamic production of molecular species that comprise the phage during infection [15] (Figure 1A right). This model was used to computationally predict the infection outcome of phage genome modifications [16], [17]. Separately, host *E. coli* metabolism has been most comprehensively modeled using Flux Balance Analysis (FBA), which uses linear optimization of an objective function to solve a system of steady-state mass balance ODEs [18]. FBA-based models have expanded to account for essentially all of the known metabolic functionality in *E. coli* (Figure 1A upper left) [19]–[21]; these models capture growth rates and nutrient exhaustion as well as the impact of genome perturbation and evolutionary outcomes over time [22]–[24].

Two previous extensions of the *E. coli* FBA and T7 ODE models have attempted to encode some dependence of viral replication on host state. One effort was based on the *E. coli* FBA model, with metabolic reactions added to describe production of MS2 virions [25], thus demonstrating the fundamental translation of viral composition to host metabolic terms (the analogous translation for T7 is denoted in Figure 1A, lower left). The implemented FBA objective function assumed that the host optimized all of its resources toward viral production immediately upon infection, resulting in an overprediction of phage production. The other modeling effort added a set of correlations between the host growth rate and the availability of replication machinery for T7 processes [26], improving the model's predictions (Figure 1A upper right) to the T7 ODE model.

Both of these efforts strongly suggest that a comprehensive, detailed effort to integrate the host and virus into a single computational model will significantly advance our understanding of viral infection in its metabolic context. Ideally such an effort would build on previous work with this host-virus system, despite the different ODE and FBA modeling techniques. Integration of FBA and ODE-type models sets the flux values for a subset of reactions using available kinetic rate equations [27], providing a conceptual framework for combining the host and viral models as depicted in Figure 1A.

Here we present an integrated model that is based equally on *E. coli* FBA and the T7 ODEs. It includes a mathematical description of metabolic reactions and demand introduced by the virus, as well as a simulation algorithm that facilitates interaction between the two models throughout the entire course of infection. Our integrated modeling approach enables us to predict phage production changes as the host nutritional environment shifts, and provides insight into the underlying limiting factors in T7 infection.

## Results

### An Integrated Model of E. coli and T7 Infection

Our integration of the T7 ODEs and *E. coli* FBA (Figure 1A) began with a set of additions to each of the individual models. The *E. coli* FBA model stoichiometric matrix required new reactions to describe the routing of host precursors and energy towards viral synthesis. One reaction was constructed for the synthesis of each viral species represented in the ODE model: mRNA and protein for each of 59 viral genes, viral genome synthesis, and a reaction enforcing the recharge of nucleotide monophosphates (NMPs) released from host genome degradation (123 total reactions; Figure 1B and Methods). The T7 ODEs required one ‘production only’ reaction rate equation for each of the 123 phage reactions that consume the host metabolites that were added to the host FBA; the net concentration change rate for each molecular species in the original T7 ODEs consisted of production minus consumption terms. However, only the production rate term constrained the stoichiometric reaction in the FBA.

Furthermore, predictions based on the T7 ODEs are valid for a single infection cycle only, and lysis has not been modeled because knowledge of the proteins involved is still insufficient to inform a meaningful representation [28]. As a result we constrained the scope of the integrated model to one single infection cycle.

Next, we expanded the integrated-FBA approach beyond its original capacity to handle the viral demand for resources when these resource demands outpaced the host production capacity. The original implementation of integrated-FBA [27] included ODEs based on central metabolism, which were informed by the environmental state and thus remained within the capacity of host metabolism without any direct communication of host limitations. In contrast, the T7 ODEs do not encode variation in the environmental conditions or the corresponding changes in the host network state's supply of metabolites. As a result, conflicts between the viral metabolite demands and host metabolite supply can arise during the simulation. We therefore encoded communication of information about host limits to the T7 ODEs. This strategy was complicated by the fact that the kinetic formulation of the T7 ODEs is largely independent of small molecule concentrations, except for the nucleotides required for T7 genome synthesis. Furthermore, FBA does not provide concentration information.

**Figure 2:**
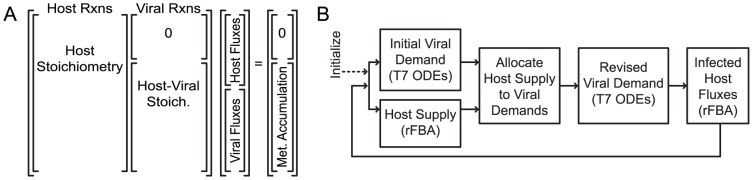
Format and method for the integrated simulation. (A) The combined host-viral form of the integrated FBA problem is a stoichiometric matrix (Stoich.) that can be considered as blocks: left, the independent host stoichiometric matrix; right, viral reactions consuming host metabolites. The combined matrix may be further organized by host metabolites that do not supply viral reactions (rows of the 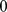 matrix in the upper right) and host metabolites that are consumed by viral reactions (rows at the bottom aligned with Host-Viral Stoich). The vector of fluxes contains host reaction rates at the top and viral reaction fluxes at the bottom to multiply properly with the host-left and viral-right organization of reactions in the stoichiometric matrix. Accumulation is allowed at the intersections of host viral metabolism (Met. Accumulation; right), but the steady-state assumption is enforced for host-only metabolites (0). A simplified flowchart (B) of the algorithm for integrated simulations, where Initialize indicates the definition of media nutritional conditions and the start of iterations across time, simulating at each integration time point the individual T7 ODEs and *E. coli* FBA, then reconciling the viral rate metabolite demand with host network state supply (Allocate). Both models are then recalculated to incorporate information on their mutual constraint (Revised Viral Demand, and Infected Host Fluxes). Update of environmental information and regulatory constraints at the initiation of each integration step (not specifically denoted on figure) further constrains the host-viral system.

Consequently, we devised a metabolite allocation-based approach to bounding reaction rates. Recognizing that the host-viral metabolic interface is the set of common metabolites used in macromolecule synthesis, we split the matrix formulation (Figure 2A) into a sum of metabolite rate vectors that represent the host supply and viral demand, where the former constrains the latter. Given a selected host flux distribution, we calculate a strict bound on viral metabolite use. Due to the lack of kinetic information about how the viral metabolic reactions contribute to the metabolite demand, we assume that all viral reactions have an equal and high affinity for precursor metabolites. After calculating rates for the viral reactions from the T7 ODEs to determine the demand for viral metabolites, we scale the rates of all reactions consuming a given metabolite by the same fraction such that total demand is brought within host supply. This method assures that while all reactions are limited evenly, no reaction is limited by a metabolite it does not consume; if amino acids are scarce but dNTPs are available, genome synthesis can proceed but translation cannot.

In summary, this allocation method converts the information about the host metabolic network state into constraints on the T7 ODEs. We implemented this method as part of an algorithm for T7 ODE and *E. coli* FBA integration with bidirectional information exchange and mutual constraint at each time step (Figure 2B). After initial specification of the host nutritional environment, the overall viral demand is calculated (without consideration of host limits) using the T7 ODEs, and the host capacity calculated using FBA. Host supply and viral demand are reconciled by calculating the upper bounds on viral production fluxes, after which the T7 ODEs are re-evaluated over the integration time step because metabolite limitation of one viral ODE may affect the ODE solution as a whole. Finally, the infected host flux distribution is calculated using optimization on the host metabolic network, with viral fluxes bounded exactly to constrained T7 ODE reaction rate values.

### Comparing Model and Experimental Data - Tryptone Media

**Figure 3:**
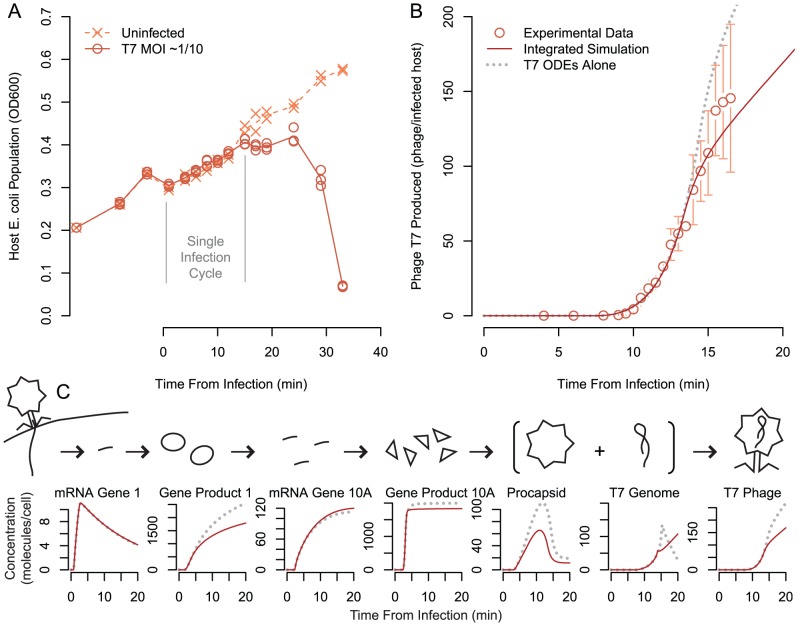
Host population and phage population time courses. (A) Dynamic time courses of experimental host population data uninfected (line is mean of n = 2) and infected cultures (line is mean of n = 3); an immediate drop in population density occurs when the solution of phage is added at 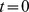, due to dilution. Initial infection multiplicity was 0.1. (B) Measured and simulated phage production per infected host in tryptone broth media (circles are mean, error bars shown are the standard deviation, n = 3). Simulation presented for the integrated model and T7 ODEs alone simulated at 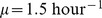. (C) Expanded comparison of the simulated concentrations of critical phage replication machinery and phage virion components compared to T7 ODEs alone. Gene Product 1 is the T7 RNA polymerase; Gene product 10A is the major capsid protein.

To validate the ability of the model to predict infection phenotypes, we observed *E. coli* infection by T7 during growth on tryptone broth. We first measured the growth of *E. coli* cultures in the presence and absence of T7 (Figure 3A). The culture is cleared within 35 minutes, representing approximately two infection cycles at 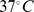.

Unfortunately, with standard OD resolution, the infected and uninfected cultures were not distinguishable from one another within the single infection cycle (Figure 3A) simulated by the model. Thus, differences in host growth rate were not a useful metric to assess the prediction performance of our computational model. We therefore returned to the traditional plaque assay-based approach to determine the number of phage produced per infected host cell during a single initial infection cycle, consistent with previous work with the T7 ODEs [15], [26] (Figure 3B). We observed rapid increases in the number of phage beginning around 10 minutes.

To compare model predictions to observations, we simulated phage production time courses under the same environmental conditions using our fully integrated model as well as the T7 ODEs alone. We found that the T7 ODEs alone substantially overpredicted the production of T7 phage over time (Figure 3B). This overprediction has been reported previously [15], [26]. The integrated model more accurately captured the phage production time course (Figure 3B), suggesting that the integrated model is limiting the production of T7 virions (detailed comparison across media given below).

To determine the cause of this limitation, we considered the model's predictions of phage production and host metabolism in more detail. We compared simulated intracellular concentrations of selected phage components for the integrated simulation to those during simulation of the T7 ODEs alone (Figure 3C). The model predicts that production of Gene Product (GP) 1 is limited at translation; GP 1 is the T7 RNA polymerase and is required to transcribe middle and late T7 genes. Despite reduced transcription capacity, sufficient mRNA for the major capsid protein (Gene 10A) is still produced. Major capsid protein production is metabolically limited at translation, and thus procapsid availability for phage assembly is decreased, resulting in fewer phage produced during late infection than predicted by the T7 ODEs alone. In the integrated simulation, although phage T7 genome is produced at the same rate as the T7 ODEs alone, it is not packaged as quickly, with a considerable fraction of the total genomes produced remaining unpackaged after assumed lysis. This excess phage T7 genome resulting from phage production limitation at the protein level is consistent with previous experimental observations [15]. The most prominent limitation by metabolism appears during the later steps of replication: mid and late gene product synthesis and genome production. In contrast, mRNA production is relatively unperturbed early in the simulation, suggesting that metabolic limitation varies in its impact over different periods during infection.

**Figure 4:**
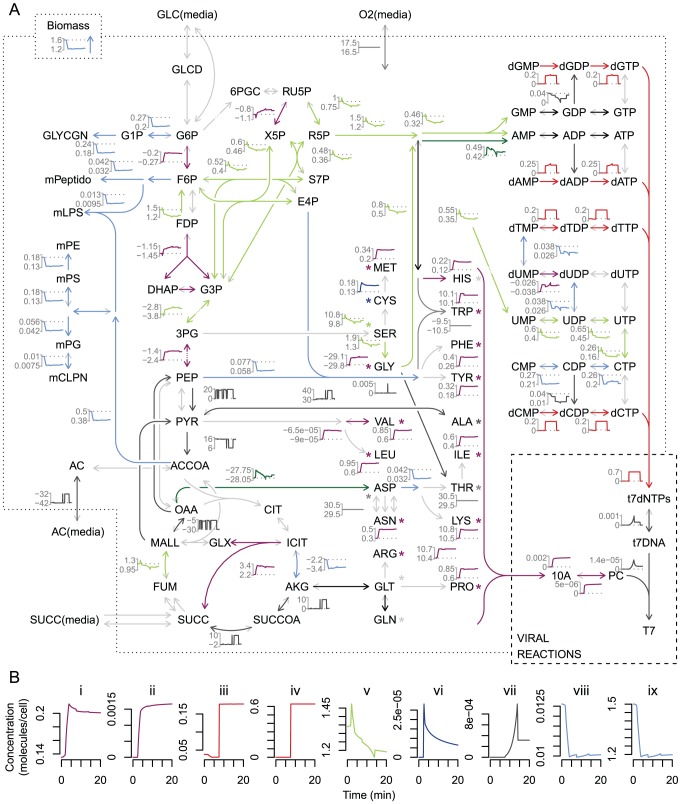
Infected host fluxes on tryptone media. (A) Flux dynamics are displayed for a subset of the metabolic network map. Arrows representing reactions and the subplots of flux through those reactions are colored according to clustering of flux dynamics. Positive flux values correspond to the reaction direction indicated by the colored arrowhead, negative flux direction is depicted with light grey barbs. Asterisks (*) represent an abbreviation of the arrow for uptake from media. Metabolite abbreviations are consistent with FBA model definition. For clustering, fluxes were treated as vectors with (1-correlation) as distance, and clustered using average hierarchical grouping with a cutoff height of 0.25. Clusters with fewer than ten members appear in black, and clusters with constant dynamics are highlighted in grey. All nonzero fluxes in any media (tryptone, glucose, succinate, and acetate) were included in the flux clustering so that cluster designation and color coding is consistent across media and figures. Maps for media other than tryptone are included in the SI. (B) Select flux dynamics expanded for clarity ordered to exemplify host flux changes driven by viral dynamics: (i) host amino acid synthesis, (ii) major viral capsid protein synthesis, (iii) host nucleotide phosphorylation, (iv) viral digestion of host genome to dNMPs, (v) purine biosynthesis, (vi) viral mRNA synthesis, (vii) viral genome synthesis, (viii) host cell envelope biosynthesis, (ix) host biomass accumulation.

After considering the phage reaction changes in the integrated simulation, we used the model to investigate the changes in host metabolism during infection. The flux-balance component of the integrated model calculates a predicted flux distribution for *E. coli* growth on tryptone in the presence and absence of phage. Essentially all of the non-zero fluxes change dynamically over time in the presence of T7; a subset of these changes are shown alongside the underlying metabolic map (Figure 4). Many metabolic reactions experienced prominent flux changes that were coordinated during infection. Dynamic coordination of fluxes in time is not particularly surprising considering the underlying network structure of constraints. However, these similarities in addition to the sheer number of total fluxes that require consideration render unaided visual inspection of infection dynamic information rather uninformative. We found it useful to cluster the flux dynamics into broad categories, which facilitate interpretation of the interesting flux patterns in central and peripheral metabolism during viral replication.

The majority of the observed flux clusters are driven by viral flux requirements (Figure 4B). The increase in amino acid synthesis and uptake corresponds in time to the synthesis of viral proteins (Figure 4Bi–ii), and similarly flux through nucleotide phosphorylation is high during the period of host genome digestion to dNMPs and viral use of dNTPs (Figure 4Biii–iv). Increased nucleotide recharge and pooling is known to occur during phage T7 replication, due at least in part to interactions between phage gene products and host metabolic enzymes [2]. Some complex host flux dynamics result from multiple viral resource interactions (Figure 4Bv–vii); flux towards nucleotides first increases during rapid early viral mRNA production, and then decreases as viral genome synthesis occurs, corresponding to the presence of large quantities of nucleotides.

Flux towards host membrane components and cofactors decreases as the ability of the host to synthesize biomass is reduced by the viral draw on components (biomass flux decrease before 5 min) and energy (biomass flux decrease between 5 and 10 mins during dNTP recycling) (Figure 4Bvii–ix; light blue). This cluster is the largest of the nonzero flux clusters across and within media, and the sharp decrease in flux within 5 min represents the shutdown in processes that are not required by the virus. Interestingly, this shutdown is not explicitly encoded by either model and therefore represents an emergent property of the integrated model system. The detailed flux maps therefore provide potential for a deeper biological insight regarding the underlying metabolic changes that occur during viral infection.

### Comparing Model and Experimental Data - Other Media

The T7 ODEs were originally parameterized to fit data where *E. coli* grew on tryptone broth or other rich media [15]. Later work incorporated correlations between available host machinery (e.g., ribosomes) and host growth rate into the ODEs in order to account for the effect of growth rate on infection dynamics [26]. Host metabolism is encoded explicitly in our integrated host-virus model, and so instead of a given growth rate parameter, the integrated model requires only the environmental conditions as inputs.

**Figure 5:**
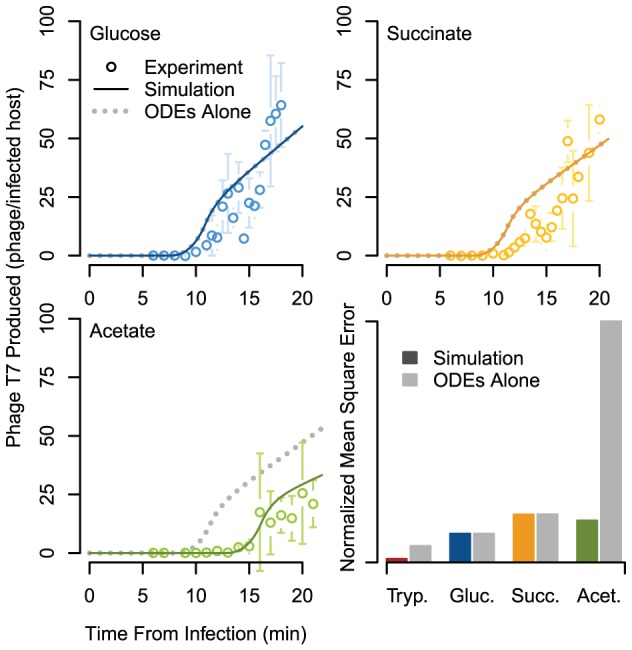
Measured and simulated phage production. Shown per infected host, across time, experiment compared to model predictions for integrated model system, and the T7 ODEs alone, on M9 minimal media with glucose, succinate, or acetate as carbon source (growth rates for T7 ODEs alone are 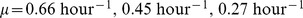, respectively). Error bars are standard deviation of n = 3. For glucose and succinate media the T7 ODEs time course is not visible because it falls directly beneath the integrated simulation line. The lower right panel quantifies the goodness of fit of the integrated simulation and the T7 ODEs alone to experimental observations using normalized mean squared error.

Unlike either individual model, the integrated model is capable of predicting the viral infection dynamics for many different culture conditions. We tested model predictions for three previously unmodeled conditions: glucose, succinate, and acetate minimal media. In each case, we measured the phage production over time (Figure 5, bottom left and top panels). For glucose and succinate media, the models produced dynamics nearly identical to each other as well as similar to the experimental data. However, for infections on acetate minimal media, the integrated model was more accurate than the T7 ODEs alone. The two predicted time courses differ because the integrated model accounts for the slow growth and nutritional limitation of *E. coli* on acetate (roughly half of the growth rate on succinate). In particular, small decreases in gene product synthesis result in delayed achievement of the thresholds necessary for phage genome replication initiation. Furthermore, all of the simulations, from both the integrated model and the ODEs alone, deviate from the typical one-step-growth phage production trajectory. This is due to the rigid description of host DNA degradation and incorporation into viral genomes in the ODEs, which was originally characterized under a single environmental condition. Quantitative comparison of our observations to the model predictions verified that tryptone simulations were the most indicative of experiment, and that the tryptone and acetate integrated model simulations outperformed those of the ODEs alone (Figure 5, bottom right panel).

We next wanted to understand how the host and viral fluxes change under these different nutrient conditions. Detailed individual media flux maps analogous to 4 are provided for glucose, succinate, and acetate media in Figures S2, S3, and S4 respectively. To generate a global evaluation of the host flux response to infection on varying media, we analyzed the aggregate similarity of the total flux distribution between pairs of media (Figure S5). Generally this comparison indicated that the flux distribution for infection during growth on acetate was very similar to the distribution during growth on succinate, while there was more divergence between the tryptone and glucose flux distributions than for any other media pair.

**Figure 6:**
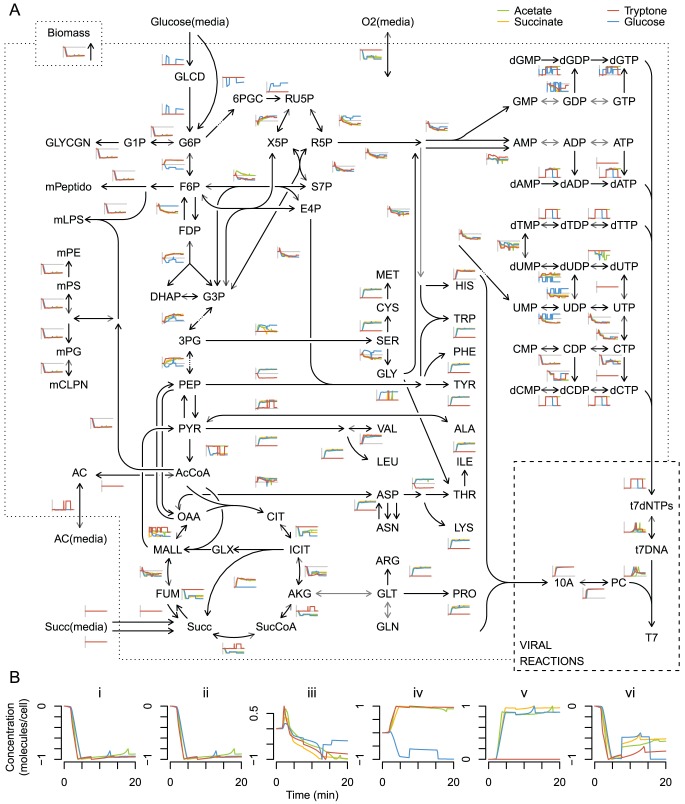
Comparing normalized infected host flux dynamics spark-lines for all four media. (A) Metabolic map and normalized flux dynamics for tryptone, glucose M9, succinate M9, and acetate M9 media. Flux values were shifted to the uninfected value (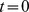), and then normalized to their maximum magnitude on each medium; zero (initial) value is indicated by a grey horizontal line. Metabolite abbreviations are consistent with FBA model definition. (B) Expansion of a selected subset of normalized fluxes. Host cell envelope synthesis (i), and biomass accumulation (ii) decrease similarity across media. Purine synthesis (iii) exhibits dynamic similarity across media. Glycolysis (iv) is observed on glucose while gluconeogenisis occurs on other media. Amino acid synthesis (v) increases on minimal media but not on amino acid-rich tryptone; and the citric acid cycle (vi) demonstrates similarity in dynamic flux change timing, but differences in scaling and direction.

Figure 6 displays the dynamic metabolic flux distribution for all four infection simulations, normalized to facilitate comparison. Of the fluxes that are non-zero in any of the media conditions, a large fraction show highly similar dynamics. These fluxes include critical biomass-related reactions such as those that contribute to membrane (Figure 6Bi–iii) or ribonuclotide biosynthesis. In some regions of the metabolic network, flux dynamics depend more on the media conditions; for example, in central metabolism the flux direction is often reversed between glucose and the other media because glycolysis is occurring rather than gluconeogenesis (Figure 6Biv). Reactions involved in amino acid synthesis also exhibit this phenomenon, as they increase in rate on all three minimal media, yet are zero on tryptone medium (Figure 6Bv), which contains amino acids. Another interesting example involves citric acid cycle activity, which is especially increased during the high energy demands of nucleotide recycling (Figure 6Bvi). One final subset, adjacent to key metabolites such as pyruvate (PYR), oxaloacetate (OAA), and succinate (SUCC), displayed erratic and rapid jumps between their extreme values, which results from equivalent optimal flux distributions calculated by FBA in highly interconnected sections of the metabolic network.

Finally, we used our model results to address the issue of host-based limitation of viral infection. Many studies assume that phage infection of *E. coli* is limited by “machinery” – the number of ribosomes, RNA polymerases, and similar factors. Another possibility is that in some cases the host metabolic rates are limiting factors; however, decoupling this limitation is difficult due to the regulation of *E. coli* protein synthesis capacity by the availability and type of nutrients [29]. We sought to compare the effects of *E. coli* machinery- or metabolic-based limitation on T7 infection, an exploration enabled by our integrated simulation which can be perturbed in ways not practical experimentally. The detailed simulation output presented in Figure 3C indicates that metabolic limitation may be more prominent for certain phage processes and during specific periods of infection. As a summary output for comparison across conditions, we chose the phage production at seventeen minutes post infection. This point is shortly after which all cultures had begun to lyse, releasing phage, and thus making the bulk quantity relevant to phage propagation across generations within a host population.

**Figure 7:**
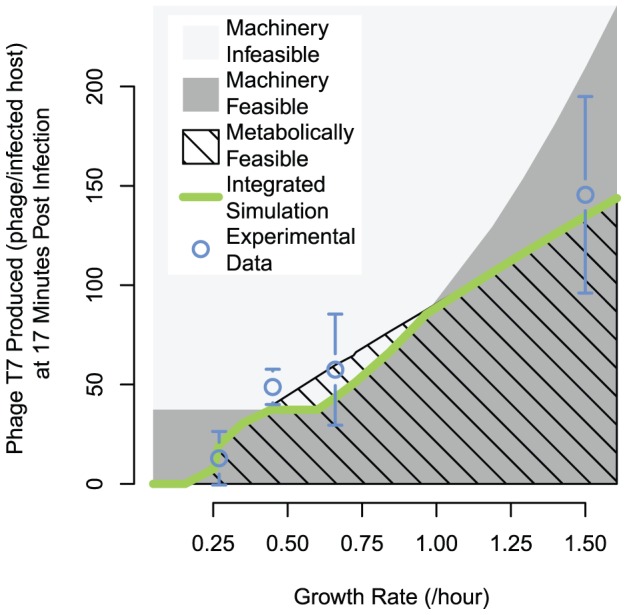
Variation in the limiting factor for phage production across host growth rates. Modeling results overlaid with experimental phage production measurements. The machinery-feasible region represents phage production values from T7 ODEs alone, with the growth rate supplied to correlations for availability of the host replication machinery; phage production values above the machinery-feasible boundary are considered machinery infeasible. The upper boundary of the metabolically feasible region was calculated using the integrated simulation, but with access to excess host replication factors, which we simulated by multiplying the host growth rate from FBA by a factor of 1.25 when it was passed to the T7 ODE host machinery correlations. Growth rate variation for calculating limitation boundaries and integrated simulation was evaluated with a set of modified flux bounds, with most growth rate sampling values simulated with both carbon and oxygen limitation, which produced essentially identical phage production predictions (resulting points lie within width of the line displayed). Error bars are standard deviation of n = 3.

The boundary representing machinery limitations is provided by evaluation of the T7 ODEs alone across varied input growth rates (Figure 7). The region that falls below the model prediction is feasible (dark gray), and everything above is not (light gray). To calculate the bounding metabolic phage production limitation, we simulated the integrated model with the modification that excess host replication machinery components were provided to the ODE model (accomplished by passing a higher host growth rate to the ODEs than that predicted by FBA). This calculation was carried out for carbon- and oxygen-limited growth at each resulting growth rate, which resulted in uniform predictions of phage production at each growth rate. Metabolic feasibility here refers to the supply of small molecule metabolites needed to build phage virions; the metabolic limit increases smoothly with host growth rate because the phage is made of a subset of the metabolites included in the host biomass reaction that represents FBA growth, and a state of host growth maximization is assumed for host supply. This context reveals the integrated model to be slightly mechanistically limited over the range of growth rates between approximately 0.4/hour and 1/hour, and more severely metabolically limited at higher and lower growth rates; however, simulations at very low growth rates do produce empty capsids, reflecting the strong repression of virion DNA production encoded in the ODEs. Metabolic limitation at high and low growth rates explains the better performance of the integrated model than the T7 ODEs alone in predicting phage production on acetate and tryptone media (Figure 3 and 5 respectively).

## Discussion

In summary, we investigated the role of host metabolism in viral infection. *E. coli* infection by T7 provided a unique opportunity to address this issue because each system had been modeled, parameterized, and tested independently. We integrated the host metabolic FBA and T7 ODE models and compared the resulting integrated model predictions with new experimental observations. We found that our integrated model was not only a better predictor of viral infection dynamics than either of the individual models for a range of experimental conditions, but also shed new insight on the interplay between virus and host during infection. Most of the active host metabolic pathways were highly impacted by the metabolic demand imposed by virion production. Moreover, we grouped and categorized these pathways by their dynamics; these groups were directly related to the timing of viral demand for key virion components.

It is commonly assumed that viral infection dynamics are predominantly limited by the amount of protein synthesis machinery in the host [15], [30]. In contrast, our results suggest that in many cases metabolic limitation is at least as severe as machinery limitation. This conclusion in turn implies that the wealth of available metabolic reconstructions may enable computational predictions on virion production even when detailed information about interaction with host macromolecules is lacking. More broadly, these results emphasize the importance of considering viral infections in the context of host metabolism.

Finally, we anticipate that models such as this integrated model may be used to rationally perturb the viral infection process by manipulating the host. The modeling and integration approaches developed here are general to a host flux-balance model and a set of viral ODEs, and by integrating the two it may be possible to predict key host metabolic factors whose absence would hamper infection, even as these factors depend on environmental conditions.

## Materials and Methods

### Bacterial Strains, Phages, Media, and Assays

The bacterial host strain used was *E. coli* K12 BW25113, and WT T7 phage (ATCC, BAA-1025-B2) was propagated according to established protocol [31]. Tryptone media contained 10 g/liter Tryptone (BD Bionutrients Bacto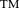 Tryptone) and 5 g/liter NaCl consistent with previous T7 work [15], [31]. M9 minimal media contained 56.4 g/liter Difco M9 Minimal salts, with added 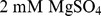 and 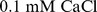; carbon sources glucose, succinate, and acetate were added at 10 mM, 15 mM, and 30 mM to media preparations, respectively. All culture experiments were conducted at 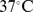 in a circulating water bath at a volume of 30 ml culture in a 250 ml flask that was magnetically stirred. Infections were at an initial MOI of 0.1 to assure hosts would only be infected once, and replicates were taken from separately infected flasks. Host population was measured as the optical density (OD) using a spectrophotometer at a wavelength of 595 nm. Phage dilution and storage was in SM phage buffer [26]. Measurements of phage titer were made by plating phage sample with 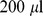 fresh bacterial culture at 1 OD from tryptone media in 3 ml tryptone broth with 0.7% agar atop tryptone broth 1% agar, and incubating the plate in an inverted position at 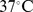 for approximately 3 hr [31].

### Phage Time Course Assays

One-step phage growth experiments were conducted consistent with published protocols [15], [26], [28]. Prior to infection, bacterial hosts grew exponentially to a total density of 0.2 OD. Pilot experiments suggested that essentially all phage absorbed into the host cells within five minutes. Therefore, after 5 minutes of infection in the initial culture flask, a sample was diluted 1000-fold in warm shaken media into another flask of the same total culture volume (30 ml) to minimize adsorption of produced phage to new hosts. At 6 and 7 minutes (time points selected as just following complete phage absorption)infected hosts were counted. To count infected hosts 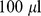 samples were transferred into ice-cold 900 ml aliquots of phage buffer, returned to ice, and plated less than 30 minutes later. At 6 and 7 minutes, as well as all other time points, 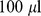 samples were transferred into room-temperature 900 ml aliquots phage buffer with 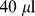 chloroform for host lysis. The chloroformed samples were incubated at room temperature for 30 minutes with periodic vortexing, then stored at 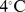 until plating, usually within an hour. Phage from lysed samples at later time points are reported normalized to the infected host count obtained by the difference of unlysed and lysed samples at 6 and 7 minutes.

### Modeling Approach

We implemented the T7 ODEs in MATLAB (R2011a The MathWorks Inc.), informed by the equations presented in the initial publication [15] as well as the code available for the most recent version [26]. The T7 ODEs were originally compared to phage production data at 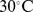 having been simulated using parameters measured at either 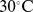 or 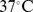 [15], [26]. The published flux bounds and regulatory rules of FBA correspond to *E. coli* growth at 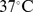, and therefore for consistency the T7 parameters were modified to 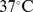 where necessary (Table S5). This modification included kinetic parameters and promotor strengths to maintain prediction constancy with the proportion of phage gene products produced [32], (Tables S4). A stiff solver (ode15s) was used for all solutions of T7 ODEs, as required by discontinuous rate definition equations.

The regulatory-FBA model reaction equations and metabolites are iMC1010v2 [33], with the minor change that a few reversible reactions were reversed for pathway direction consistency. Media definitions for simulated M9 minimal were consistent with past publications and tryptone media was approximated as amino acids (Table S1); the short time of T7 infection meant that media components were in excess for all simulations with growth rate limitations resulted from flux bound constraints. Some regulatory rules were updated to permit growth on rich media (Table S2). Flux bounds were mostly consistent with previous publications, with the exception of the relevant set used during growth on tryptone amino acids that were fit using growth rates we collected (Figure S1).

Phage stoichiometry reactions were included in the FBA system (Figure 1B), one for each gene”s mRNA and each gene product, as well as for phage genome synthesis and a reaction accounting for degraded host genome dNMP recycling to dNTPs. Included in these reactions are the precursor small molecules that make up each final macromolecule, as well as the energy required for transcription or translation. The FBA host biomass reaction energy requirements are typically phrased in terms of ATP only; to be consistent, the GTP used for energy in phage production processes is included in the reaction stoichiometry as ATP, and the energy requirements for the T7 DNA helicase, which is known to use dTTP preferentially [34] for energy, were also converted to ATP. A full list of assumptions and references for generating phage stoichiometry reactions is in Table S3.

We added a production rate equation consisting of only the positive terms from the net rate equation for each molecular species in the original T7 ODE model, to bound the forward-only reaction fluxes in FBA. Furthermore, another ODE was added to account for the fraction of the host genome material remaining for degradation. A set of input arguments to the T7 ODEs was also introduced to pass limits on one or more of the production rates. If a production rate was limited, its value is accounted for in the net rate equation. Implicit in this implementation is the assumption that if an mRNA or gene product is degraded, the components are not available to metabolism during infection [35].

The code used in preparation of this article is available at 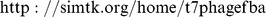.

### Integrated Simulation Algorithm

A simplified flowchart of the integrated simulation algorithm is shown in Figure 2B. The FBA and ODE numerical simulations interacted at every 10 seconds of simulation time. Since host lysis is not modeled by the T7 ODEs, there is not a single logical exit criterion for the simulation. Thus the simulation is run for a set time length slightly greater than what is expected to be the productive duration of infection. A text expansion of the integrated simulation algorithm flowchart shown in (Figure 2B) follows, with further detail presented in (Text S1 and Figure S6):

1. **Specification:** Define media composition of nutrient concentrations, including those that are replenished (often O2) and those that are exhaustible (usually carbon source).
2. **Initialization, Host:** Determine steady regulation state and growth rate in media, set all media to replenished, and run sequential rFBA simulations until convergence. Set initial time point host regulation state and pass growth rate to T7 ODEs.
3. **Initialization, Virus:** Evaluate T7 ODE host growth rate correlations to set model parameters for host machinery availability. Set initial concentration state of viral ODEs to 0, except for the variable representing host genome for degradation, which is set from growth rate correlations.
4. **Initial Viral Demand:** Evaluate T7 ODEs without any limits imposed for initial estimates of the amount of resources the virus will request from host metabolism. Many viral sub time steps are made within integration time step as determined by ODE solver.
5. **Host Supply:** Set flux bounds based on environmental availability, and regulatory rules referencing environment and host state. Evaluate host linear programming problem (maximize biomass flux in this case) to determine host resources feasibly available to viral reactions.
6. **Allocate Host Supply To Viral Demands:** Distribute metabolites to viral fluxes and set production reaction rate bounds (see expanded section that follows).
7. **Revised Viral Demand:** Evaluate T7 ODEs with production reaction limits. Many viral sub time steps within integration time step as determined by ODE solver.
8. **Infected Host Fluxes:** Set viral reaction fluxes in FBA vector to net viral production rate averaged over integration time step, and evaluate combined linear programming problem (maximize biomass flux) to arrive at overall flux distribution.
9. **Update States:** Consumption and excretion to/from the environment, flux distribution values, viral concentrations. Return to 4 or exit.

### Metabolite Distribution

Because the T7 ODE kinetic rates do not depend on small molecule concentrations, we bound the phage macromolecule production rates themselves to host production capacity. The method to determine rate limits relies first on an initial ‘viral demand’ which is based on an evaluation of the T7 ODEs without applied limits over the integration time step. Implementation of this strategy takes advantage of the divided matrix formulation of the problem shown in Figure 2A. We further split the matrix (detail in supplement) into summed terms representing the small metabolites provided by the host 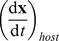, and those consumed by the viral production fluxes 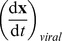. In the resulting relationship, shown in Eq. 1 (consistent with convention of FBA intake to organism being negative flux), 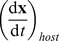 is the solution of the typical host FBA problem neglecting biomass exchange, taking advantage of the fact that host biomass is composed of a superset of the small metabolites consumed by viral reactions. The simplified form of this relationship is enabled by allowance of metabolite accumulation at the intersection of host and viral reactions.
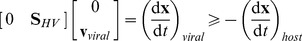
(1)


Once a feasible host flux distribution is selected (by solving for a ‘host supply’ flux distribution), Eq. 1 provides a simple relation that must be obeyed by viral production flux rates in order to assure a solution exists to the combined host viral metabolic problem. The method devised to select a vector of maximal viral fluxes or rates (to pass to T7 ODEs) is detailed in the supplement, but essentially allows the maximal evenly scaled flux through viral reactions consuming any given metabolic precursor. For example, allowing full production of viral DNA even if amino acid availability is limiting protein synthesis, yet restricting both if a shared reactant such as ATP is limiting.

## Supporting Information

Figure S1 Time courses of uninfected *E. coli* growth on tryptone, succinate, glucose and, acetate. Each time course was fit by a simple exponential (dotted) as well as using dynamic FBA (solid line), where initial conditions were determined by the first experimental measurement.
(EPS)

Figure S2 Infected host fluxes on glucose M9 minimal media. (A) Flux dynamics are displayed for a subset of the metabolic network map. Arrows representing reactions and the subplots of flux through those reactions are colored according to clustering of flux dynamics. Positive flux values correspond to the reaction direction indicated by the colored arrowhead, negative flux direction is depicted with light grey barbs. Asterisks (*) represent an abbreviation of the arrow for uptake from media. Metabolite abbreviations are consistent with FBA model definition. For clustering, fluxes were treated as vectors with (1-correlation) as distance, and clustered using average hierarchical grouping with a cutoff height of 0.25. clusters with fewer than ten members appear in black, and clusters with constant dynamics are highlighted in grey. All nonzero fluxes in any media (tryptone, glucose, succinate, and acetate) were included in the flux clustering so that cluster designation and color coding is consistent across media and figures. (B) Select flux dynamics expanded for clarity ordered to exemplify host flux changes driven by viral dynamics: (i) host amino acid synthesis, (ii) major viral capsid protein synthesis, (iii) host nucleotide phosphorylation, (iv) viral digestion of host genome to dNMPs, (v) purine biosynthesis, (vi) viral mRNA synthesis, (vii) viral genome synthesis, (viii) host cell envelope biosynthesis, (ix) host biomass accumulation.
(EPS)

Figure S3 Infected host fluxes on succinate M9 minimal media. (A) Flux dynamics are displayed for a subset of the metabolic network map. Arrows representing reactions and the subplots of flux through those reactions are colored according to clustering of flux dynamics. Positive flux values correspond to the reaction direction indicated by the colored arrowhead, negative flux direction is depicted with light grey barbs. Asterisks (*) represent an abbreviation of the arrow for uptake from media. Metabolite abbreviations are consistent with FBA model definition. For clustering, fluxes were treated as vectors with (1-correlation) as distance, and clustered using average hierarchical grouping with a cutoff height of 0.25. clusters with fewer than ten members appear in black, and clusters with constant dynamics are highlighted in grey. All nonzero fluxes in any media (tryptone, glucose, succinate, and acetate) were included in the flux clustering so that cluster designation and color coding is consistent across media and figures. (B) Select flux dynamics expanded for clarity ordered to exemplify host flux changes driven by viral dynamics: (i) host amino acid synthesis, (ii) major viral capsid protein synthesis, (iii) host nucleotide phosphorylation, (iv) viral digestion of host genome to dNMPs, (v) purine biosynthesis, (vi) viral mRNA synthesis, (vii) viral genome synthesis, (viii) host cell envelope biosynthesis, (ix) host biomass accumulation.
(EPS)

Figure S4 Infected host fluxes on acetate M9 minimal media. (A) Flux dynamics are displayed for a subset of the metabolic network map. Arrows representing reactions and the subplots of flux through those reactions are colored according to clustering of flux dynamics. Positive flux values correspond to the reaction direction indicated by the colored arrowhead, negative flux direction is depicted with light grey barbs. Asterisks (*) represent an abbreviation of the arrow for uptake from media. Metabolite abbreviations are consistent with FBA model definition. For clustering, fluxes were treated as vectors with (1-correlation) as distance, and clustered using average hierarchical grouping with a cutoff height of 0.25. clusters with fewer than ten members appear in black, and clusters with constant dynamics are highlighted in grey. All nonzero fluxes in any media (tryptone, glucose, succinate, and acetate) were included in the flux clustering so that cluster designation and color coding is consistent across media and Figures S2, S3, and S4. (B) Select flux dynamics expanded for clarity ordered to exemplify host flux changes driven by viral dynamics: (i) host amino acid synthesis, (ii) major viral capsid protein synthesis, (iii) host nucleotide phosphorylation, (iv) viral digestion of host genome to dNMPs, (v) purine biosynthesis, (vi) viral mRNA synthesis, (vii) viral genome synthesis, (viii) host cell envelope biosynthesis, (ix) host biomass accumulation.
(EPS)

Figure S5 Similarity of flux dynamics within a media condition compared to similarity across different media conditions. Correlation of each pair of fluxes within a metabolic class (Y), plotted against the correlation between a single flux between pair of media (Y-X). T, tryptone; G, glucose; S, succinate; A, acetate. Flux dynamics were treated as vectors to calculate the Pearson correlation. Only pairs that include one non-zero flux value were considered; for constant-constant pairs of flux dynamics a correlation of 1 was assigned, and for constant-varying pairs a correlation of 0 was assigned. Individual flux correlations were aggregated as density for plotting (darker as more dense), using kernel smoothing with a grid of 80 points and a bandwidth of 0.25. The density shading scale is not comparable between pairs. Similarity, as measured by positive correlation, of flux distributions indicated by high density on the right of the axis, and similarity within a single media indicated as density in upper regions. Centered density on either axis indicates dissimilarity, or lack of correlation. Panels highlighted with green are the highly similar flux dynamic distribution pair acetate succinate, most alike to the analysis of media with itself for reference, bounded in black. Panels highlighted with a blue border (glucose to succinate or acetate) are largely similar with some uncorrelated fluxes. Panels highlighter with red border (tryptone to any of the minimal media) are largely dissimilar. All media pairs display a large correlated fluxes because viral fluxes which are constrained by the T7 ODEs, which have dynamics that are similar except for scale across media. Inverse correlation within media and metabolic class potentially arises from the arbitrary directionality assigned to reversible reactions in the FBA definition.
(EPS)

Figure S6 Detailed algorithm used for integrated simulation of *E. coli* FBA and T7 ODEs. Solid lines are each integration time step, from beginning to end of iteration top to bottom. Shaded boxes are stored states describing model time course. Dotted lines completed for initialization. Dashed line connecting Viral Demand and Host Supply is needed or not needed depending on the optimization method being implemented in the latter. Full expansion of steps in Text S1.
(EPS)

Table S1 FBA simulation media definitions.
(PDF)

Table S2 List of FBA rules relaxed for rich media growth.
(PDF)

Table S3 Assumptions and references for construction of phage stoichiometry reactions.
(PDF)

Table S4 Table of major T7 ODEs genome definition update.
(PDF)

Table S5 T7 ODEs parameter updates, values, and references.
(PDF)

Text S1 Expanded methods detail.
(PDF)
