# Supplementary material for: Determining Host Metabolic Limitations on Viral Replication via Integrated Modeling and Experimental Perturbation
Source: PLoS Comput Biol. 2012 Oct 18;8(10):e1002746. doi: 10.1371/journal.pcbi.1002746 (PMC3475664; doi:10.1371/journal.pcbi.1002746)
Supplement: Table S1 — FBA simulation media definitions. (PDF) [file pcbi.1002746.s007.pdf]

Table S1. FBA simulation media definitions.

FBA Metabolite	Tryptone	Glucose	Succinate	Acetate
H20	55 mM	55 mM*	55 mM*	55 mM*
CO2	15 mM*	15 mM*	15 mM*	15 mM*
PI	15 mM*	15 mM*	15 mM*	15 mM*
H	10 mM*	10 mM*	10 mM*	10 mM*
SLF	10 mM*	10 mM*	10 mM*	10 mM*
O2	10 mM*	10 mM*	10 mM*	10 mM*
NH3	10 mM	10 mM	10 mM	10 mM
GLC	-	10 mM	-	-
SUCC	-	-	10 mM	-
AC	-	-	-	10 mM
ASP	5.7 mM	-	-	-
MET	1.6 mM	-	-	-
THR	3.7 mM	-	-	-
ILE	3.3 mM	-	-	-
SER	5.8 mM	-	-	-
LEU	6.3 mM	-	-	-
GLU	16.4 mM	-	-	-
TYR	1.6 mM	-	-	-
PRO	8.0 mM	-	-	-
PHE	2.7 mM	-	-	-
GLY	3.06 mM	-	-	-
HIS	1.6 mM	-	-	-
ALA	3.92 mM	-	-	-
LYS	4.9 mM	-	-	-
CYS	0.5 mM	-	-	-
ARG	1.9 mM	-	-	-
VAL	5.7 mM	-	-	-
TRP	0.5 mM	-	-	-
ASN	0.5 mM	-	-	-

Asterisk (*) indicates nutrients replenished to the given concentration at each time step.
