# Supplementary material for: Determining Host Metabolic Limitations on Viral Replication via Integrated Modeling and Experimental Perturbation
Source: PLoS Comput Biol. 2012 Oct 18;8(10):e1002746. doi: 10.1371/journal.pcbi.1002746 (PMC3475664; doi:10.1371/journal.pcbi.1002746)
Supplement: Table S2 — List of FBA rules relaxed for rich media growth. (PDF) [file pcbi.1002746.s008.pdf]

Table S2. List of FBA Rules Relaxed for Rich Media Growth.

Rxn	Original Rule
BCAAUP1R	b0401
BCAAUP2R	b0401
BCAAUP3R	b0401
ARGA	b2818
ARGB	b3959
ARGCR	b3958
ARGDR	b3359
ARGE1	b3957
ARGFR	b0273
ARGIR	b4254
ARGHR	b3960
SERC1	b0907
DAPB	b0031
LYSA	b2838
DEOB1R	b4383
DEOC	b4381
DEOD1R	b4384
DEOD2R	b4384

Changes relative to rules in iMC1010v2 [1]. Rules relaxed indicates regulatory boolean expression altered to TRUE.

References

1. Covert MW, Knight EM, Reed JL, Herrgard MJ, Palsson BO (2004) Integrating high-throughput and computational data elucidates bacterial networks. Nature 429: 92–96.
