# Supplementary material for: Determining Host Metabolic Limitations on Viral Replication via Integrated Modeling and Experimental Perturbation
Source: PLoS Comput Biol. 2012 Oct 18;8(10):e1002746. doi: 10.1371/journal.pcbi.1002746 (PMC3475664; doi:10.1371/journal.pcbi.1002746)
Supplement: Table S3 — Assumptions and references for construction of phage stoichiometry reactions. (PDF) [file pcbi.1002746.s009.pdf]

**Table S3. Assumptions and References for construction of phage stoichiometry reactions.**

| Viral mRNA production reactions | |
|---|---|
| Nucleotide stoichiometry for all 60 T7 bacteriophage mRNAs from genome sequence and to date publications. | GenBank (NC 001604) |
| Each added NTP was assumed to produce one pyrophosphate during polymerization. | [1] |
| **Viral Gene Product production reactions** | |
| Amino acid stoichiometry for all 60 T7 bacteriophage proteins. | GenBank |
| tRNA charging was assumed to hydrolyze 1 ATP to AMP and pyrophosphate | [1] |
| Aminoacyl-tRNA binding was assumed hydrolyze 1 GTP to GDP | [1] |
| tRNA translocation during translation was assumed to hydrolyze 1 GTP to GDP | [1] |
| Polymerization initiation was assumed to hydrolyze 1 GTP to GDP | [1] |
| **Host genome degradation and recycling reaction** | |
| Nucleotide stoichiometry for T7 bacteriophage and E. coli genomes | GenBank (U00096) |
| During host genome degradation dNMPs were assumed to be produced in proportion to their content in the E. coli genome. | - |
| During viral genome production dNTPs were assumed to be used in proportion to their content in the T7 genome and converted into 1 viral dNTP. | - |
| **T7 Genome synthesis reaction** | |
| Nucleotide stoichiometry for T7 bacteriophage genome | GenBank |
| Each added dNTP was assumed to produce one pyrophosphate during polymerization | [1] |
| DNA helicase was assumed to hydrolyze 2 ATP to ADP per base pair of DNA unwound | [2] |
| Lagging strand synthesis: RNAmer primers 4 nucleotides long were assumed to hydrolyze 2 high energy phosphate bonds per polymerized NTP (8 high energy phosphate bonds total) | [1,3] |
| Lagging strand synthesis: Okazaki fragments were assumed to be 3,500 bases long on average | [3] |
| Lagging strand synthesis: 2 high energy phosphate bonds were hydrolyzed to regenerate the NAD used in ligation | [1] |
| Proofreading: Error rate in T7 DNA polymerase was assumed to be 10 percent for dATP, 5 percent for dTTP, 1 percent for dGTP and dCTP. These were counted as additional A,T,G,C incorporated into the genome and then hydrolyzed to dNMPs | [1,4] |
| In all cases high energy phosphate bonds were accounted for as ATP being hydrolyzed to ADP. | - |

Changes relative to rules in iMC1010v2 [5]. Rules relaxed indicates regulatory boolean expression altered to TRUE.

# References


1. Neidhardt FC, Ingraham JL, Schaechter M (1990) Physiology of the bacterial cell. a molecular approach. Sinauer Associates Inc.

2. Lee SJ, Richardson CC (2010) Molecular Basis for Recognition of Nucleoside Triphosphate by Gene 4 Helicase of Bacteriophage T7. Journal of Biological Chemistry 285: 31462–31471.

3. Engler MJ, Richardson CC (1983) Bacteriophage T7 DNA replication. Synthesis of lagging strands in a reconstituted system using purified proteins. The Journal of biological chemistry 258: 11197–11205.

4. Rappaport H (2004) The fidelity of replication of the three-base-pair set adenine/thymine, hypoxanthine/cytosine and 6-thiopurine/5-methyl-2-pyrimidinone with T7 DNA polymerase. Biochemical Journal 381: 709.

5. Covert MW, Knight EM, Reed JL, Herrgard MJ, Palsson BO (2004) Integrating high-throughput and computational data elucidates bacterial networks. Nature 429: 92–96.
