# Supplementary material for: Determining Host Metabolic Limitations on Viral Replication via Integrated Modeling and Experimental Perturbation
Source: PLoS Comput Biol. 2012 Oct 18;8(10):e1002746. doi: 10.1371/journal.pcbi.1002746 (PMC3475664; doi:10.1371/journal.pcbi.1002746)
Supplement: Table S4 — Table of major T7 ODEs genome definition update. (PDF) [file pcbi.1002746.s010.pdf]

Table S4. Table of Major T7 ODEs Genome Definition Update.

Parameter	Explanation of Change	Value	Units	Reference
$n_{gp10A,PC}$	GP10A stoichiometry in procapsid. Updated to reflect combined inclusion of GP10A (415) and GP10B (16), treated as single species because T7 ODEs lack description of small percentage programed frameshift distinguishing between these proteins which are of very similar length and stoichiometry.	431	$\frac{molecules}{procapsid}$	[1]
$Gene10B$	GP10B genomic element removed during inclusion in 10A (see $n_{gp10A,PC}$)	-	-	-
$n_{gp6.7,T7}$	GP6.7 DNA completion or ejection protein. Not in virion in original T7 ODEs.	18	$\frac{molecules}{phage}$	[1]
$n_{gp7.3,T7}$	GP7.3 Adsorption and/or ejection protein. Not in virion in original T7 ODEs.	33	$\frac{molecules}{phage}$	[1]
$n_{gp8,T7}$	GP8 head tail connector. Not changed from T7 ODEs.	12	$\frac{molecules}{phage}$	[1]
$n_{gp11,T7}$	GP11 tail protein. Stoichiometry updated.	12	$\frac{molecules}{phage}$	[1]
$n_{gp14,T7}$	GP14 core and/or ejected protein. Stoichiometry updated.	10	$\frac{molecules}{phage}$	[1]
$n_{gp15,T7}$	GP15 core and/or ejected protein. Stoichiometry updated.	8	$\frac{molecules}{phage}$	[1]
$n_{gp16,T7}$	GP16 core and/or ejected protein. Stoichiometry updated.	4	$\frac{molecules}{phage}$	[1]
$\phi_{4.7,4.3,4c,3.8,2.5,1.6,1.5}$	Promotor weighting	0.01	Relative Units	See SI text
$\phi_{6.5}$	Promotor weighting	0.05	Relative Units	See SI text
ϕ_{9}	Promotor weighting	0.2	Relative Units	See SI text
$\phi_{13,17}$	Promotor weighting	0.1	Relative Units	See SI text
$\eta_{T\phi}$	T7 terminator efficiency	0.85	Fraction	See SI text

Changes relative to genome used previously [2], other changes are based on any nucleotide/protein updates in current GenBank sequence for T7 the impact of which is negligible with respect to simulation predictions.

References

1. Kemp P, Garcia LR, Molineux IJ (2005) Changes in bacteriophage T7 virion structure at the initiation of infection. Virology 340: 307–317.

2. Endy D (1997) Development and application of a genetically-structured simulation for bacteriophage T7. Ph.D. thesis.
