# Supplementary material for: Determining Host Metabolic Limitations on Viral Replication via Integrated Modeling and Experimental Perturbation
Source: PLoS Comput Biol. 2012 Oct 18;8(10):e1002746. doi: 10.1371/journal.pcbi.1002746 (PMC3475664; doi:10.1371/journal.pcbi.1002746)
Supplement: Table S5 — T7 ODEs parameter updates, values, and references. (PDF) [file pcbi.1002746.s011.pdf]

# References


1. García LR, Molineux IJ (1995) Rate of translocation of bacteriophage T7 DNA across the membranes of Escherichia coli. Journal of Bacteriology 177: 4066–4076.

2. Bremer H, Yuan D (1968) Chain growth rate of messenger RNA in Escherichia coli infected with bacteriophage T4. Journal of Molecular Biology 34: 527–540.

3. Chamberlin M, Ring J (1973) Characterization of T7-specific ribonucleic acid polymerase. The Journal of biological chemistry 248: 2235.

4. Yamada Y, Whitaker PA, Nakada D (1974) Early to late switch in bacteriophage T7 development: functional decay of T7 early messenger RNA. Journal of Molecular Biology 89: 293–303.

5. Dalbow D, Young R (1975) Synthesis time of beta-galactosidase in Escherichia coli B/r as a function of growth rate. Biochemical Journal 150: 13.

6. Lee SB, Bailey JE (2002) Analysis of growth rate effects on productivity of recombinant Escherichia coli populations using molecular mechanism models. Biotechnology and Bioengineering 79: 550–557.

7. Tabor S, Huber HE, Richardson CC (1987) Escherichia coli thioredoxin confers processivity on the DNA polymerase activity of the gene 5 protein of bacteriophage T7. The Journal of biological chemistry 262: 16212–16223.

8. Donlin MJ, Johnson KA (1994) Mutants affecting nucleotide recognition by T7 DNA polymerase. Biochemistry 33: 14908–14917.

9. Son M, Watson RH, Serwer P (1993) The direction and rate of bacteriophage T7 DNA packaging in vitro. Virology 196: 282–289.

10. Kemp P, Garcia LR, Molineux IJ (2005) Changes in bacteriophage T7 virion structure at the initiation of infection. Virology 340: 307–317.

11. Ionel A, Velázquez-Muriel J, Luque D (2011) Molecular rearrangements involved in the capsid shell maturation of bacteriophage T7. Journal of Biological Chemistry 286: 234–242.

12. Sadowski P, Kerr C (1970) Degradation of Escherichia coli B deoxyribonucleic acid after infection with deoxyribonucleic acid-defective amber mutants of bacteriophage T7. Journal of Virology .

**Table S5. T7 ODEs Parameter updates, values, and references.**

| Parameter | Explanation of Change | Value | Units | Reference |
|---|---|---|---|---|
| $k_{T7,transloc}$ | Phage T7 translocation rate. All rates were measured at 37C. Second and third rates are determined by E. coli and T7 RNA polymerase transcription rates respectively (see below). | [ 141 , 55 , 400 ] | bp/sec | [1–3] |
| $k_{Ec,RNAP}$ | E. coli RNA polymerase transcription rate. Measured at 37C. | 55 | nt/sec/RNAP | [2] |
| $k_{T7,RNAP}$ | T7 RNA polymerase transcription rate. Measured at 37C to be 2x faster than 200 nt/s/RNAP found at 30C. | 400 | nt/sec/RNAP | [3] |
| $k_{d,T7mRNA}$ | T7 mRNA decay rate. Functional decay of early T7 mRNA found to be 6.5min at 30C, converted to rate, conservatively not adjusted for temperature since data is for early transcripts. | 0.001 | /sec | [4] |
| $k_{RIBO}$ | Specific translation rate or ribosomes. Measured at 37C. | 42 | nt/sec/ribo | [5] |
| $k_{d,pro}$ | T7 Protein Decay Rate. Adjusted from 2.8E-5 /sec at 30C to 37C using Arrhenius approximation. | $3.92 \times 10^{-5}$ | /sec | [6] |
| $C_{repDNA}$ | T7 DNA Replication critical threshold of gp1 binding by gp3.5. Stated as arbitrarily set in T7v2.5 code. | $5 \times 10^{-7}$ | M | - |
| $k_{T7,DNAP}$ | T7 DNA Replication rate. Found to be over 300 bp/sec/polymerase at 37C using techniques in Tabor et al. 1987. | 475 | $\frac{bp}{sec \cdot polymerase}$ | [7] |
| $K_{M,DNA}$ | DNA polymerase Km. Measured at 37C. | 8668 | nt/cell | [8] |
| $k_{T7,pack}$ | T7 DNA Packaging rate. Adjusted from 0.702 /min at 30C to 37C using Arrhenius approximation. | 0.983 | /min | [9] |
| $C_{nuc,PC}$ | Procapsid nucleation concentration. Changed to avoid numerical solver error, to single multiple of procapsid stoichiometry (see below). Original value used was for phage P22, understanding of T7 assembly mechanism recently updated. | 431 | $\frac{molecules}{cell}$ | [10, 11] |

Continued...

**Table S5. Continued.**

... Continued

| Parameter | Explanation of Change | Value | Units | Reference |
|---|---|---|---|---|
| $k_{d,EcDNA}$ | Host genome degradation rate. As per original T7 ODEs, assuming rate consistent with 7.5 to 15 min degradation, 0.85 of host genomic material degraded, and $n_{HG}$ the equivalent number of host genomes from correlation. Factor results from $\frac{\left(4.655\times10^{6}bp\right)\frac{2nt}{bp}}{7.5min\frac{60s}{min}}0.85$ | $n_{HG}\left(1.76\times10^{4}\right)$ | $\frac{nt}{s}$ | [12] |
| $t_{d,EcDNA}$ | Host genome degradation period. Original limits 7.5 to 15 min measured for 37C, allowed to continue past 15 min if degradation limited by metabolic interaction. | $> 7.5$ | min | [12] |
