# Supplementary material for: Determining Host Metabolic Limitations on Viral Replication via Integrated Modeling and Experimental Perturbation
Source: PLoS Comput Biol. 2012 Oct 18;8(10):e1002746. doi: 10.1371/journal.pcbi.1002746 (PMC3475664; doi:10.1371/journal.pcbi.1002746)
Supplement: Text S1 — Expanded methods detail. (PDF) [file pcbi.1002746.s012.pdf]

Text S1: Method Detail – Determining Host Metabolic Limitations on Viral Replication via Integrated Modeling and Experimental Perturbation

Elsa W. Birch[1], Nicholas A. Ruggero[1], Markus W. Covert[2,*]
1 Chemical Engineering, Stanford University, Stanford, California, USA
2 Bioengineering, Stanford University, Stanford, California, USA
∗ **E-mail: mcovert@stanford.edu**

Introduction

Here we include detailed information about the integrated model, as well as further experimental and computational data.

Simulation of environmental conditions

The media definitions used for simulations are given in Table S1. The three minimal media are well-defined; the Tryptone media composition is based on the BD Bionutrients^{TM} Technical Manual. While most metabolite media components are considered exhaustible, some metabolites are present in excess, which is simulated by returning to a preset concentration at every FBA time step.

Certain modifications had to be made in the integrated model to account for different media conditions. For example, our experiments were performed at 37°C, but the original T7 ODEs were produced to model a 30°C environment. We therefore modified some of the parameters, as listed and explained in Table S5. Equilibrium binding constants were assumed not to change with temperature. Parameters not listed in Table S5 are consistent with previous implementations [1].

Furthermore, the original FBA regulatory rules identified in [2] had never been optimized for growth on rich media, and the model was therefore very limited in its ability to simulate reasonable growth rates. We therefore relaxed several of these rules to allow smooth growth on media containing only amino acid carbon sources in the combination present in our representation of tryptone. A list of relaxed regulatory rules is given in (Table S2).

Some of the FBA uptake bounds were determined by fitting dynamic rFBA simulations to experimental measurements in the absence of virus. Experimental data for host growth in the different media are shown, together with fitted exponentials, in Figure S1. The measured growth rates were: tryptone $\mu = 1.5$ hour^{-1}, glucose $\mu = 0.66$ hour^{-1}, succinate $\mu = 0.45$ hour^{-1}, and acetate $\mu = 0.27$ hour^{-1}. Also shown are dynamic regulatory FBA simulations of host growth, the growth rates of which were fit via flux bound changes to the growth rate found by exponential fit. These fits were produced by estimation of key flux bounds, as follows:

for oxygen,

$$v_{min,O2} = -17\frac{mmol\ rxn}{gdcw \cdot hour},$$

for glucose

$$v_{min,GLC} = -7.3\frac{mmol\ rxn}{gdcw \cdot hour},$$

for succinate

$$v_{min,SUCC} = -10.5\frac{mmol\ rxn}{gdcw \cdot hour},$$

for acetate
$$v_{min,AC} = -13 \frac{mmol\ rxn}{gdcw \cdot hour},$$
and for all amino acids
$$v_{min,AA} = -30 \frac{mmol\ rxn}{gdcw \cdot hour}.$$
We include the integrated simulation output for glucose (Figure S2), succinate (Figure S3), and acetate (Figure S4) M9 minimal media in flux maps analogous to that presented for tryptone in Figure 4 of the main text. Color coding of flux arrows by cluster is consistent across figures, including tryptone.

Finally, to generate a more global evaluation of the host flux response to infection on varying media, we analyzed the aggregate similarity of the total flux distribution between pairs of media (Figure S5). Overall, the flux distribution for infection during growth on acetate was very similar to the distribution during growth on succinate (green), largely because both carbon sources require gluconeogenesis for growth. The flux distributions for growth on glucose (blue) and tryptone (red) were more divergent because glucose is utilized by glycolysis and tryptone is unique among the four media for containing several amino acid carbon sources.

Updates to the T7 ODEs and FBA model

A number of additions were made to the T7 ODEs to reflect scientific advances and understanding subsequent to the model's original publications. Minor stoichiometry updates were made to reflect the most recent understanding of T7 virion composition, Table S4. Stoichiometric multipliers are rounded up to integer values from value in citation. We also changed a subset of the promoter values for class II and III genes to adjust the ratio of gene product production to more closely correspond to the fraction in virion production and observed experimentally [3], Table S4. We did not alter the early gene promotors or those for *E. coli* RNA polymerase in order to preserve critical feedback and inhibitory interactions. Table S5 lists the parameter changes in detail.

The FBA model also had to be altered to account for viral-related reactions. The general reaction forms shown in Figure 1B of the main text were constructed using the assumptions in Table S3. For each gene in the T7 genome, a viral mRNA and viral protein production reaction was added to FBA.

Degradation and recycling of the host genome by T7 are described as a single reaction accounting for the conversion of degraded host genome dNMPs through host pathways to directly produce viral dNTPs. A single reaction was used instead of multiple reactions (e.g., a separate degradation of host genome to dNMTs and uptake of dNTPs) in order to prevent the host from using these nucleotides, consistent with the pooling observed during T7 infection.

Another single reaction accounting for the synthesis of phage T7 genomes accounts for energy in both strands of synthesis as well as for the energy and metabolites involved in proofreading.

Integrated Simulation Algorithm

The integrated simulation algorithm presented in Figure 2 of the main text is detailed in Figure S6, and each step expanded below. Implementation was in MatLab and the code is available from the repository at http : //simtk.org/home/t7phagefba.

One note about the overall simulation algorithm is that the calculation of host supply and allocation across viral reactions is implemented without having first established whether the metabolite resources requested by the viral reactions exceed the possible host supply. This approach enforces our assumption that the virus is limited to the metabolic supply of the uninfected host state. If host supply calculation and resource allocation were completed only in the case that viral reaction metabolite use over-constrained

the host problem, then the assumption of viral supply would be switched mid simulation – from viral maximization (which may not violate the bounds of the host but may exceed the metabolites available from a host biomass optimized flux distribution), to viral production being constrained by the host optimized metabolic state. This approach therefore maintains consistent application of our assumptions across time steps.

Specification (Start)

For each individual simulation run environmental perturbations are implemented in the media definition file, which specifies the media components and their concentrations in the initial Environment State.

Initialization (1)

Simulations are initialized using dynamic regulatory FBA simulation replenishing each media component. The steady state biomass production predicted by FBA is passed to the growth rate correlations given in the main and supplemental text and code of You *et al.* 2002 T7v2.5 [1].

Evaluate Viral Demand (2)

In this step, the T7 ODEs are evaluated as written with no limits applied using the viral state as initial condition concentrations and evaluated from t to $t + \Delta t_{integration}$, where $\Delta t_{integration}$ is the time step. The viral state is a vector of intercellular molecular concentrations separate from the FBA variables, which is stored at each time point of integration. The T7 ODE numerical solution output for the time period is in the format of concentration changes over time, and must be converted to a flux equivalent. Using the production-only term of the reaction rates associated with host fluxes $\frac{dP_j}{dt}$, the associated concentration variable changes over the time step can be directly averaged to flux unit values

$$\mathbf{v}_{request,ave} = \frac{([P_{j,t=(t+\Delta t_{int})}] - [P_{j,t=t}])}{\Delta t_{integration}}. \tag{1}$$

Several steps of the viral ODEs run between FBA steps, and we choose the maximum demand from all viral time steps. An average demand over all time steps was determined to be unsuitable because it unduly restrained viral production during the many time steps of rapid (and discontinuous) rate change based on phage genome translocation.

The maximum demand is evaluated as

$$\mathbf{v}_{request,max} = max_{(across\ t)} \left(\frac{\mathrm{d}[P_j]}{\mathrm{d}t} \right) \tag{2}$$

where the numerical approximation of rate

$$\frac{\mathrm{d}[P_j]}{\mathrm{d}t} \approx \frac{([P_{j,t=(t+\Delta t_{ODE})}] - [P_{j,t=t}])}{\Delta t_{ODE}} \tag{3}$$

is made for rates at each ODE evaluation time step.

This method to approximate viral demand has the potential disadvantage that the maximal flux value will lead to an over-request of some viral reactions, leading to an allocation of resources to this reaction that it will not use but is unavailable to other viral reactions. To mitigate this effect, we hold the integration time step below 15 sec.

Set Host Flux Bounds (3)

Next, we determine the bounds on all fluxes in the FBA model based on regulatory rules, environmental conditions, and viral demand. The Boolean regulatory relationships and substrate uptake bounds (except for those discussed earlier in the SI) were evaluated as in [2], based on time step $t_{integration}$ and biomass. These set the bounds on host reactions used in all subsequent linear optimization steps. Accumulated metabolites, which are allowed at the intersection of host and viral metabolism, based on the assumption that the developmental process of viral replication do violate steady state over single time-steps, and may be used in subsequent steps by the host (longer term accumulation is not observed). Mathematical accounting for the accumulation of metabolites as well as their consumption in later steps is directly analogous to exchange with the media, even in so far as concentrations are stored on a biomass independent basis. The model source code contains conversion factors between metabolite rates ($\mathbf{b}$) and accumulated concentrations, which are stored in the units equivalent to a media concentration. Viral demand bounds were obtained as defined in the previous section.

Optimization for Host Supply (4)

With the bounds determined, we used FBA to determine the host-virus flux distribution. FBA requires an objective function to calculate an optimal flux distribution. A common assumption in choosing an objective function is that the cell culture maximizes its growth rate, subject to the defined constraints [4]. For the very brief course of T7 phage infection (10-15 minutes), we assumed the state of host metabolism as set by the presence of enzymes remained relatively similar to the uninfected state. This enabled us to retain biomass maximization as the objective function. For comparison purposes, we also evaluated a strategy wherein the objective was to maximize viral reaction fluxes - to a maximum of $\mathbf{v}_{request}$. The simulation results for both objective functions were indistinguishable, due to the optimization of host after viral constraint (8).

We made two further modifications to the general FBA approach to enable modeling of T7 infection. First, T7 breaks down the host chromosome and the resulting free nucleotides are recycled and used for virion synthesis. Chromosome breakdown is therefore a major introduction of metabolites into the host metabolic network that are being modified, rather than synthesized from media sources. To account for chromosome breakdown, we added a reaction to enable host nucleotide recycling. In order to make the viral dNTPs available during the metabolite distribution step, we maximized recycling in a separate and subsequent optimization step. A separate recycling step is required because it is a draw on host energy resources would therefore not occur as a result of host biomass optimization. Furthermore, this approach approximates the concentration effects that are known to occur due to kinetics and T7 encoded gene product interactions with host metabolic enzymes, which lead to rapid host recharge of its own degraded genome nucleotides [5].

Our second modification to FBA involved the production of certain metabolites by the virus that could be used by the host. Most of the metabolite transfer in the simulation flows from host to virus, but some viral reactions include a return of metabolites to the host (e.g., ADP is produced when the virus uses ATP, or dNMPs result from proofreading of mistakes in T7 genome synthesis). These fluxes of metabolic resources into the host can then be used in other reactions after viral bounds are strictly set (8), but do not contribute during the calculation of the host supply upper bound.

Allocate Metabolites Among Viral Reactions (5)

Because the T7 ODE kinetic rates do not depend on small molecule concentrations, we bound phage macromolecule production rates themselves to host production capacity. The method to determine rate limits relies first on the 'initial demand' calculated as described above. All reaction rates consuming a given metabolite are then scaled to the availability of that metabolite. Implementation of this strategy takes advantage of the matrix formulation of the FBA problem, and splitting the combined Host-Viral

stoichiometric matrix as shown in main text Figure 2a into $\mathbf{S}_H$ the host stoichiometry, $\mathbf{S}_{HV}$ host-viral stoichiometry of host metabolite consumption by viral reactions Eq. 4.

$$\begin{bmatrix} \mathbf{S}_H & \mathbf{S}_{HV} \\ 0 & \mathbf{S}_V \end{bmatrix} \begin{bmatrix} \mathbf{v}_{host} \\ \mathbf{v}_{viral} \end{bmatrix} = \frac{\mathrm{d}\mathbf{x}}{\mathrm{d}t} \tag{4}$$

$$\begin{bmatrix} \mathbf{S}_H & \mathbf{S}_{HV} \end{bmatrix} \begin{bmatrix} \mathbf{v}_{host} \\ \mathbf{v}_{viral} \end{bmatrix} = \frac{\mathrm{d}\mathbf{x}}{\mathrm{d}t} \tag{5}$$

It should be noted that a matrix representation of the combined problem might include $\mathbf{S}_V$ which is viral metabolite stoichiometry, presumably predominantly macromolecules. It is not strictly necessary provided mass conservation is enforced by the ODEs, and so $\mathbf{S}_V$ is henceforth neglected Eq. 5. By splitting the matrix and flux vector components, we represent the product of the combined metabolic system instead as a sum of metabolite rate vectors Eq. 6 which are the net metabolites produced by the host, $\left(\frac{\mathrm{d}\mathbf{x}}{\mathrm{d}t}\right)_{host}$, and net small molecule metabolite consumption by viral reactions, $\left(\frac{\mathrm{d}\mathbf{x}}{\mathrm{d}t}\right)_{viral}$.

$$\begin{bmatrix} \mathbf{S}_H & 0 \end{bmatrix} \begin{bmatrix} \mathbf{v}_{host} \\ 0 \end{bmatrix} + \begin{bmatrix} 0 & \mathbf{S}_{HV} \end{bmatrix} \begin{bmatrix} 0 \\ \mathbf{v}_{viral} \end{bmatrix} = \frac{\mathrm{d}\mathbf{x}}{\mathrm{d}t}$$

$$\left(\frac{\mathrm{d}\mathbf{x}}{\mathrm{d}t}\right)_{host} + \left(\frac{\mathrm{d}\mathbf{x}}{\mathrm{d}t}\right)_{viral} = \frac{\mathrm{d}\mathbf{x}}{\mathrm{d}t} \tag{6}$$

For a host flux distribution, $\mathbf{v}_{host}$, optimized towards a biomass production, the typical FBA formulation includes a biomass exchange reaction which renders $\left(\frac{\mathrm{d}\mathbf{x}}{\mathrm{d}t}\right)_{host}$ a steady-state vector of zeros and therefore trivial. However, if we consider the same maximized flux distribution without biomass exchange (denoted *) then this $\left(\frac{\mathrm{d}\mathbf{x}}{\mathrm{d}t}\right)^*_{host}$ is a positive metabolite rate vector representing the net small molecule precursors available for general macromolecular synthesis at that metabolic state. This metabolite rate can therefore be used to constrain viral metabolite consumption, for which we also assume that some accumulation can occur at the metabolite intersections of host and virus based on the developmental nature of virion replication Eq. 7.

$$\left(\frac{\mathrm{d}\mathbf{x}}{\mathrm{d}t}\right)_{host} + \left(\frac{\mathrm{d}\mathbf{x}}{\mathrm{d}t}\right)_{viral} = \frac{\mathrm{d}\mathbf{x}}{\mathrm{d}t} \geqslant 0 \tag{7}$$

which, abbreviating $\frac{\mathrm{d}\mathbf{x}}{\mathrm{d}t}$ as $\mathbf{b}$ simplifies to:

$$\mathbf{b}_{viral} \geqslant -\mathbf{b}^*_{host}. \tag{8}$$

We are left with the requirement only that the net viral metabolites $\left(\frac{\mathrm{d}\mathbf{x}}{\mathrm{d}t}\right)_{viral}$ not be less than $\left(\frac{\mathrm{d}\mathbf{x}}{\mathrm{d}t}\right)^*_{host}$ as shown in Eq. 8, consistent with convention of FBA intake to organism being negative flux).

Once a feasible host flux distribution is selected (Host Supply Step), this provides a simple relation that must be obeyed by viral production flux rates in order to assure a solution exists to the combined host viral metabolic problem. The method devised to select a vector of maximal viral fluxes/rates (to pass to T7 ODEs) is detailed below. For the sake of space, $\frac{\mathrm{d}\mathbf{x}}{\mathrm{d}t}$ is abbreviated $\dot{\mathbf{x}}$ in the following algorithm description. The inputs are the bounding metabolite-rate vector $\dot{\mathbf{x}}_b = -\left(\frac{\mathrm{d}\mathbf{x}}{\mathrm{d}t}\right)_{host}$, and the requested reaction rates from the unlimited ODEs $\mathbf{v}_{ode} = \mathbf{v}_{request}$. The values, vectors that are updated each iteration are denoted with a subscript i

1. Accept inputs $\dot{\mathbf{x}}_b$ and $\mathbf{v}_{ode}$. Set $\mathbf{v}_{i,ode} = \mathbf{v}_{1,ode} = \mathbf{v}_{ode}$, and $\dot{\mathbf{x}}_{i,b} = \dot{\mathbf{x}}_{1,b} = \dot{\mathbf{x}}_b$

2. Initialize variables used only within algorithm: The vector of allowed viral fluxes $\mathbf{v}_a = 0$ initialized to zero, the maximum multiplier of the requested viral fluxes $c_{i,max} = c_{1,max} = 1$ is initially unity.

3. Find the maximum c_i such that $\mathbf{S}_{HV}\mathbf{v}_{i,ode}c_i \geqslant \dot{\mathbf{x}}_b$, and subject to $0 \leqslant c_i \leqslant c_{i,max}$.

4. Update allowed viral flux vector, element-wise addition $\mathbf{v}_a = \mathbf{v}_a + \mathbf{v}_{i,ode} c_i$.
5. Update $\dot{\mathbf{x}}_{i+1,b} = \dot{\mathbf{x}}_{i,b} - \mathbf{S}_{HV} \mathbf{v}_{i,ode} c_i$ to account for resources allotted to viral fluxes this step.
6. Remove from subsequent iterations the viral fluxes that consume a resources that has been exhausted. For any element j where $(\dot{\mathbf{x}}_{i+1,b})_j = 0$, then for all elements k for which $(\mathbf{S}_{HV})_{j,k} < 0$ set $(\mathbf{v}_{i,ode})_k = 0$
7. Update maximum fraction $c_{i+1,max} = c_{i,max} - c_i$
8. If ($i > 1$ AND $c_i = 0$) OR $\mathbf{v}_{i+1,ode} = 0$ exit, returning $\mathbf{v}_a$, no greater viral flux possible, all reactions limited by at least one reactant.
9. Else, update $i = i + 1$ return to step 3.

After exit $\mathbf{v}_a$ is the vector of maximum allowed viral reaction rates through production reactions.

Evaluate Final Viral Demand (6)

Once the maximum bounding rates for each viral reaction j have been determined, $\mathbf{v}_{a,j} = r_{j,prod,limit}$, these limits are applied to production rates for viral metabolites as follows:

1. Evaluate the production rate $r_{j,prod}$ for viral species j, which is the positive kinetic term(s) representing synthesis from metabolic precursors. Also evaluate remaining consumption terms of origional kinetic rate equation, the sum of which is $r_{j,cons}$
2. If a limit has been passed and $r_{j,prod} > r_{j,prod,limit}$ then reassign $r_{j,prod} = r_{j,prod,limit}$
3. Update and return $\frac{dC_j}{dt} = r_{j,prod} + r_{j,cons}$ used in the T7 ODEs and for viral concentration output, and the production only pseudo concentration $\frac{dP_j}{dt} = r_{j,prod}$ used in the constraint of viral reactions in the host-viral FBA problem.

The final concentration change over $\Delta t_{integration}$ is then passed to determine the infected host state as well as to update the Viral State of concentrations.

Constrain Host-Viral Reaction Fluxes (7)

After the viral demand reaction rates have been determined as host compatible values, the corresponding fluxes in FBA can be constrained. To constrain the host problem to the final infected state, we set flux bounds of host-viral reactions to the average production rates determined in the evaluation of final viral demand, $\mathbf{v}_{max} = \mathbf{v}_{min} = \mathbf{v}_{ave}$, where:

$$\mathbf{v}_{ave} = \frac{([P_{j,t=(t+\Delta t_{int})}] - [P_{j,t=t}])}{\Delta t_{integration}}. \tag{9}$$

The average conversion of viral reactions to fluxes is used here because it enforces conservation of mass, in contrast to the maximum request calculation used in previous steps to allow the virus access to the high instantaneous rates of metabolite use if possible. All host flux bounds are set as previously determined.

Optimization for Infected Host (8)

Following viral constraint, the host flux distribution is still underdetermined, and so maintaining the previous assumption that host pathways remain in the state of maximal host biomass production over the course of infection, the objective function evaluated after viral reactions are constrained is optimization of host biomass production. Finally after the infected host flux distribution is determined the host and environment state can be updated according to the established relationships and another iteration of the integrated simulation proceeds from step (2).

References

1. You L, Suthers P, Yin J (2002) Effects of Escherichia coli physiology on growth of phage T7 in vivo and in silico. Journal of Bacteriology 184: 1888.

2. Covert MW, Knight EM, Reed JL, Herrgard MJ, Palsson BO (2004) Integrating high-throughput and computational data elucidates bacterial networks. Nature 429: 92–96.

3. Hopper JE, Ko G, Young ET (1975) Comparative analysis of the in vivo and in vitro expression of bacteriophage T7 messenger RNAs during infection of Escherichia coli. Journal of Molecular Biology 94: 539–554.

4. Orth JD, Thiele I, Palsson BO (2010) What is flux balance analysis? Nature Biotechnology 28: 245–248.

5. Qimron U, Tabor S, Richardson CC (2010) New Details about Bacteriophage T7-Host Interactions. Microbe 5: 117.
